# Nanoporous Carbon from Oil Palm Leaves via Hydrothermal Carbonization-Combined KOH Activation for Paraquat Removal

**DOI:** 10.3390/molecules27165309

**Published:** 2022-08-19

**Authors:** Sirayu Chanpee, Napat Kaewtrakulchai, Narathon Khemasiri, Apiluck Eiad-ua, Pornsawan Assawasaengrat

**Affiliations:** 1 Department of Chemical Engineering, School of Engineering, King Mongkut’s Institute of Technology Ladkrabang, Ladkrabang, Bangkok 10520, Thailand; 2 KUbiomass Laboratory, Kasetsart Agricultural and Agro-Industrail Product Improvement Institute, Kasetsart University, Bangkok 10900, Thailand; 3 National Nanotechnology Center (NANOTEC), National Science and Technology Development Agency, 111 Thailand Science Park, Paholyothin Road, Klong Nueng, Klong Luang, Pathum Thani 12120, Thailand; 4 College of Materials Innovation and Technology, King Mongkut’s Institute of Technology Ladkrabang, Ladkrabang, Bangkok 10520, Thailand

**Keywords:** nano-porous carbons, oil palm leaves, chemical activation, adsorption, paraquat

## Abstract

In this study, nano-porous carbon was completely obtained from oil palm leaves (OPL) by hydrothermal pretreatment with chemical activation, using potassium hydroxide (KOH) as an activating agent. Potassium hydroxide was varied, with different ratios of 1:0.25, 1:1, and 1:4 (C: KOH; *w*/*w*) during activation. The physical morphology of nano-porous carbon has a spongy, sponge-like structure indicating an increase in specific surface area and porosity with the increasing amount of KOH activating agent. The highest specific surface area of OPL nano-porous carbon is approximately 1685 m^2^·g^−1^, with a total pore volume of 0.907 cm^3^·g^−1^. Moreover, the OPL nano-porous carbon significantly showed a mesoporous structure designed specifically to remove water pollutants. The adsorptive behavior of OPL nano-porous carbon was quantified by using paraquat as the target pollutant. The equilibrium analyzes were explained by the Langmuir model isotherm and pseudo-second-order kinetics. The maximum efficiency of paraquat removal in wastewater was 79%, at a paraquat concentration of 400 mg·L^−1^, for 10 min in the adsorption experiment. The results of this work demonstrated the practical application of nano-porous carbon derived from oil palm leaves as an alternative adsorbent for removing paraquat and other organic matter in wastewater.

## 1. Introduction

Oil palm leaves (*Elaeis guineensis Jacq*.) have traditionally been derived from renewable raw resources in Southeast Asian nations such as Malaysia, Indonesia, and Thailand. One of the most common types of agricultural waste, particularly in areas where oil palms are farmed, is oil palm leaves. The management of this waste is a major issue in the industry. Open burning is the traditional method of disposing of this waste. It creates smoke, lowers soil fertility and has long-term accumulative impacts on the ecosystem of neighboring countries. This method has been banned due to its environmentally hazardous impacts. The amount of oil palm leaves waste that is generated after harvesting process has increased in recent years. Therefore, the study of palm oil leaves residue as a feedstock for nano-porous carbon should be investigated. This research demonstrated the raw material’s vast availability as a source of carbon [1,2]. Nonetheless, oil palm leaves’ residues are lignocellulosic solids made up of 30–40% neutral detergent fiber, which is an efficient carbon source owing to its composition of cellulose, hemicellulose, and lignin, all of which may be converted into carbon compounds.

Porous carbon materials are a carbon-rich product with a large porosity produced by the thermal conversion of biomass, such as Chinese fan palm [3], hazelnut shells, empty fruit bunch palm shells [4], bamboo shoot shells [5], maize stalks [6], corn stover, rice husk, peanut shells [7], and palm leaves [8]. Organic pollutant adsorption capacity on porous carbons, however, is determined by the kind of biomass, organic pollutants, and activation conditions. The porous structure of the different generated biochar, with a relatively high specific surface area, demonstrated enhanced toxin uptake, and the porous structure of porous carbon is highly advantageous for absorbing polluted water. Mohamad et al. reported the use of porous carbon derived from oil palm leaves for glycosyl removal from wastewater [9]. Abdulrhman et al. also shown that the application of activated carbon derived from Sabal palms for methylene blue adsorption [1]. Angel et al. reported on the use of the biomass from *Dioscorea rotundata* and *Elaeis guineensis* for the removal of chromium (VI) [10]. However, these materials usually have no regular morphology and a low, non-porous surface area, and their performance is restricted by the structure and composition of the raw materials. Recently, the application prospects for biomass carbon can be significantly increased by utilizing structural features. For instance, nano-porous carbon material has great advantages in the field of adsorption, where the surface area promotes mass transfer during the adsorption process. Thus, it is highly feasible and meaningful to prepare biomass carbon with a regular morphology and structure to remove pollutants from water.

Over the last decade, agricultural by-products, which are available in nature, have been identified as promising raw materials for production of porous carbons. As previously stated, numerous thermochemical conversion strategies for porous carbon synthesis have been extensively researched. The creation of nanomaterials in hydrothermal synthesis may occur over a wide range of temperatures, from room temperature to high temperatures. To control the morphology of the materials that are to be prepared, either low-pressure or high-pressure conditions could be used, depending on the vapor pressure of the main composition in the reaction hydrothermal treatment (HT). This can be used to control the morphology of the prepared materials. HT and carbonization processes are usually applied to produce carbons such as charcoal, activated carbon, and carbon fiber, which enhanced carbon content using devolatilization, resulting in porosity. These processes are widely used to create carbons such as charcoal, activated carbon, and carbon fiber, which increases carbon content through devolatilization and results in porosity. In a closed system, hydrothermal treatment, also known as the wet pyrolysis method for carbon generation, was carried out at temperatures between 150 and 250 °C [11]. Subcritical water combines with lignocellulosic polymers in the HT process, resulting in solid carbon via simultaneous processes (hydrolysis, dehydration, decarboxylation, polymerization, and aromatization) [12]. As a result, carbon generation by carbonization or dry pyrolysis is often carried out at temperatures ranging from 400 to 1100 °C in the absence of oxygen. However, by adjusting experimental factors, the relative qualities of carbon products may be adjusted (reaction temperature, holding time, and heating rate). It is important to use a cost-effective approach and increase productivity when producing NPC from a variety of biomass precursors. Traditionally, the pyrolysis carbonization process was applied to prepare activated carbons. Recently, the use of hydrothermal carbonization for the conversion of a wide variety of biomass into carbon materials has been reported [13,14]. Hydrothermal carbonization method uses the subcritical water to convert the wet/dry biomass to the hydrochar with a high oxygenated functional group content. Generally, the hydrochar products of hydrothermal carbonization would be further activation-treated to form nano-porous carbon. The relatively low operation temperature and no need for dry biomass mean that the hydrothermal carbonization of biomass is an environmentally friendly way to prepare low-cost nano-porous carbon. The main distinction between these two procedures is the presence of activating chemicals. Physical activation was achieved by employing physical reagents (steam, carbon dioxide, or partial O_2_ gas) to partially oxidize the carbon precursor at high temperatures of 700–1000 °C. Chemical activation uses substances such as sodium hydroxide (NaOH), potassium hydroxide (KOH) [15], zinc chloride (ZnCl_2_), and phosphoric acid [16,17] as the activating agent (H_3_PO_4_). However, some recent studies showed that employing KOH as an activating agent significantly increased the development of pore structures with larger surface areas in porous carbons.

The contamination of pesticides in water is a significant environmental topic that threatens both the ecosystem and public health. Paraquat (1,1-dimethyl-4,4-bipyridinium dichloride, C_12_H_14_Cl_2_N_2_) is one of the most frequently used herbicides for improving agricultural crop production around the world, owing to its great weed-killing efficiency and low cost. It is, however, exceedingly hazardous to humans and may pollute water resources (rivers and groundwater). Pesticide use has been predicted to gradually rise. Paraquat contamination in food and water supplies can represent a significant hazard to human health, even at small concentrations, because of the damage it can cause to the lung, kidney, and liver [18,19,20,21,22].

Several techniques for removing pesticides from water have been published in the literature. According to the existing literature, there are two primary ways to reduce or remove paraquat in water: (I) photocatalysis [23,24], the photo-Fenton method, chemical/electrochemical oxidation, aerobic degradation [25], and (II) adsorption by porous materials [26,27], clay, and porous silica zeolite [28]. Due to its ease of use, low cost, low energy consumption, high efficiency, and economic advantages, adsorption using porous materials is probably the most-used way of removing pesticides from water. Other methods are costly, possibly harmful to the environment and living organisms, and hard to implement.

The objective of this study was to prepare oil palm leaves with a comparatively higher surface area, porosity, porous size and carbon content, to be used as an adsorbent for paraquat in wastewater. The OPL with the highest surface area was further evaluated for carbon content at hydrothermal carbonization temperatures of 800 °C and the chemical agent KOH with different weight ratios of 1:0.25, 1:1, and 1:4 (C: KOH, *w*/*w*). The OPL with the highest surface area, porosity, porous size and carbon content was applied to the adsorption of paraquat in wastewater. The adsorption performance was obviously investigated, including removal efficiency. Equilibrium isotherm, kinetic and thermodynamics studies were also performed. The regeneration of nano-porous carbon on the adsorption performance was studied. A comparative analysis was conducted with other studies to evaluate the performance of the prepared OPL. The study will help to extend the application of abundantly available OPL waste and achieve the sustainable development goals through waste management and paraquat in wastewater.

## 2. Materials and Methods

### 2.1. Materials

Oil Palm leave (OPL), collected from oil palm plantations in Surat Thani province, Thailand, were crushed and sieved into approximate sizes of 0.5–3 mm. Hence, OPL was chosen as a raw biomass feedstock to produce nano-porous carbons. The ultimate analysis (including C, H, N, and O) of OPL feedstock is demonstrated in Table 1. The chemical compositions, such as potassium hydroxide (KOH) high-purity-grade (85%), laboratory-grade, were supplied from Carlo Erba reagents, (France). High-purity-grade (99.99%) nitrogen was used in an experiment. Deionized (DI) water was used in the experiments throughout this work.

### 2.2. Synthesis of Nanoporous Carbon

**Figure 1:**
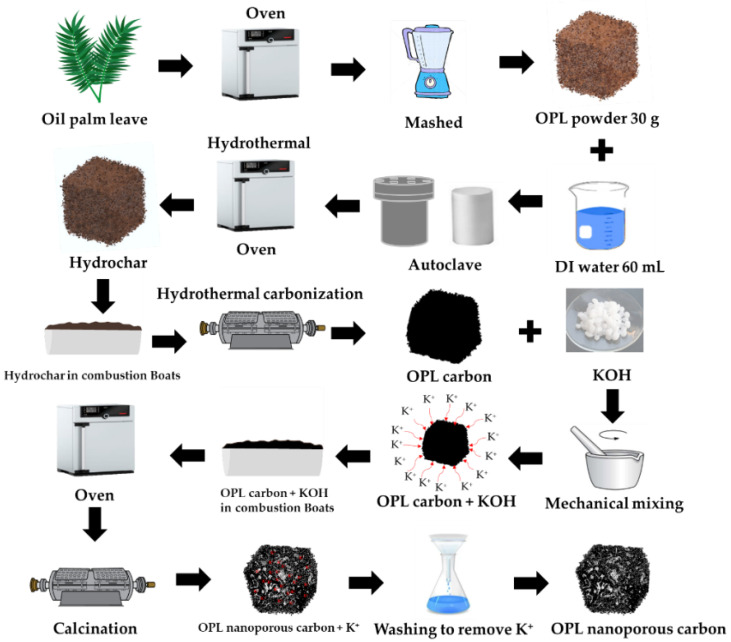
The step synthesis procedure of nano-porous carbon materials derived from oil palm leaves via hydrothermal carbonization and KOH activation.

The overall preparation of the nano-porous carbon samples in this work is schematically depicted in Figure 1. The step synthesis procedure of OPL nano-porous carbon was conducted via hydrothermal carbonization and KOH activation. Step 1: The raw OPL feedstock was washed with DI water and was then dried at 105 °C in the hot-air oven. The dried OPL was ground into powders using a grinder machine. Step 2: 30 g of the powdery OPL was mixed with 60 mL of DI water and placed into a Teflon-lined stainless-steel autoclave for the HTC process. Step 3: The hydrothermal reaction was performed at a temperature of 200 °C and time of 12 h. Step 4: Subsequently hydrochar was dried at 105 °C for overnight. The hydrochar sample obtained from an optimum hydrothermal condition was selected for further carbonization experiments. In this way, hydrothermal carbonization (HTC) provides environmental, social and economic benefits for biomass conversion process. Moreover, the HTC-combined carbonization enhanced the resulting carbon yield [3]. Step 5: The hydrochars were placed in combustion boats, brought into a horizontal tube furnace for carbonization at 800 °C for 1 h, using a heating rate of 10 °C min^−1^ under N_2_ flow of 0.2 L min^−1^, and then naturally cooled to ambient temperature. The optimum carbon sample obtained from the hydrothermal carbonization was selected for activation. The as-prepared carbon was mechanically mixed with KOH with the different weight ratios of 1:0.25, 1:1, and 1:4 (C: KOH, *w*/*w*). The carbon-to-KOH sample was heated in an oven at 105 °C for 12 h. Subsequently, the carbon was activated in a horizontal tube furnace at 800 °C for 1 h using a heating rate of 10 °C min^−1^ under N_2_ gas flow of 0.2 L min^−1^. The nano-porous carbon product was selected for the removal of paraquat. OPL nano-porous carbon was designated as OPL−KOH−800−1:0.25, OPL−KOH−800−1:1, and OPL−KOH−800−1:0.25, respectively. For comparison, the hydrothermal at 200 °C for 12 h was designated as OPL−HT−200−12. The hydrochar hydrothermal carbonized at 800 °C without KOH activation was also prepared and designated as OPL−HTC−800.

### 2.3. Characterization

#### 2.3.1. Morphology

The surface morphology was observed by field emission scanning electron microscope (FEI, model Versa). The samples were sprinkled on a carbon tape located on steel sample holder and coated by gold sputtering to enhance electron conductivity for identification. TEM images of the materials were obtained using a JEOL JEM-3100F transmission electron microscope, operated at an acceleration voltage of 300 kV. The sample dispersed in ethanol was dropped onto the Cu grid (200 square mesh coated with carbon film) and dried at room temperature overnight prior to measurements [29].

#### 2.3.2. Surface Characteristics

Relative pore characteristics, including specific surface area and porosity, were analyzed by N_2_ adsorption–desorption analysis at −196 °C using a Quantachrome Autosorp iQ-MP-XR. The Brunauer–Emmett–Teller (BET) model was used to determine the BET surface area (S_BET_) [30]. The micropore surface area and external surface area were calculated by the V_t_ method. The pore size distribution was analyzed using the density functional theory (DFT) model. The total pore volume (V_total_) was calculated by using the Barrett–Joyner–Halenda (BJH) method at the relative pressure of 0.99. The micropore (D_mic_) and mesopore (D_mes_) size distributions were obtained by the Density Functional Theory (DFT) model. The average pore size distribution (D_average_) was calculated by Density Functional Theory (DFT) model [31].

#### 2.3.3. Functional Group

The functional group on the surface of nano-porous carbon was studied by Fourier transform infrared spectroscopy (FT-IR)-modeled Perkin Elmer Spectrum Two. The infrared absorption spectra were measured in transmission mode with a wavenumber range from 4000 to 500 cm^−1^. The samples were put into infrared platform and directly screw-impressed before performing FTIR data collection. The chemical states of oxygen and carbon on the material surface were analyzed by X-ray photoelectron spectroscopy (XPS) using the Kratos Axis Ultra DLD X-ray photoelectron spectrometer equipped with a monochromic Al Kα X-ray source (1486.7 eV) operated at 15 kV and 5 mA. Survey scans were measured at a spot size of 400 µm and a constant pass energy of 200 eV. The samples were situated on the carbon tape placed on the steel stub and substituted to a high-vacuum system (1 × 10^−8^ mbar) for 2 h before measurement. All binding energy spectra were processed by multipack software to fit the desired spectra (C1s and O1s contributions) [32].

#### 2.3.4. Crystal Structural

The chemical structural properties of nano-porous carbon samples were characterized using X-ray Diffraction technique (XRD, Rikagu Smart Lab). X-ray diffractograms of all carbon samples were achieved using Cu-Kα radiation generated at 40 kV and 30 mA in steps of 0.01° S^−1^ with a step time of 0.5 s over the range of 10° < 2θ < 80° on an X-ray diffractometer [33].

#### 2.3.5. Amorphous Structural

Raman analysis was performed on a dispersed Raman spectrometer DXR Microscope of the company Thermo Scientific (Walthamm, MA, USA). We used solid-state Nd:YAG laser (wavelength 532 nm, maximum power 10 mW), recorded from 500 to 2500 cm^−1^, as an excitation source. Grating was performed with 900 lines/mm and a 25 µm slit aperture. Measurement conditions for the samples were 10 mW laser power, 10 s acquisition time per scan, and 20 repetitions. Ten spectra were averaged from each surface. Data were processed using Omnic 9 software (Thermo Scientific, Walthamm, MA, USA) [34].

#### 2.3.6. Ultimate Analysis

Ultimate analysis reveals the elemental compositions of OPL and as-performed samples (i.e., carbon, hydrogen, and nitrogen) were determined using a carbon, hydrogen, and nitrogen (CHN) elemental analyzer (ASTM D 5373-16). The oxygen percentage was directly calculated using the difference of all elemental compositions from 100% [35].

### 2.4. Adsorption Desorption and Regeneration Experiments

#### 2.4.1. Removal Efficiency of Paraquat

A mixture of the material (0.5 g) and target solution (500 mL) was placed into a TS-520D orbital shaker-flask clamp platform (Yihder Co., Ltd., Taiwan, China) operating at a shaking speed of 200 rpm at 25 °C. The PQ concentration was determined using a T92+ Spectrophotometer UV-visible spectrophotometer (PG Instruments) (λ_max_ = 257 nm). The removal efficiency of paraquat was calculated using the following:

(1) $$\mathrm{Removal}\;\mathrm{efficiency}=\frac{C_{i}-{\;C}_{t}}{C_{i}}\;\text{×}\;100$$

where C_i_ is the initial strength of the paraquat at the time (mg·L^−1^), and C_t_ is the final concentration of paraquat solution at certain time intervals (mg·L^−1^) [36,37,38,39].

#### 2.4.2. Adsorption Capacity of Paraquat

The paraquat adsorption capacity was calculated according to the following equations [17]:

(2) $$q_{e}=\frac{\left(C_{i}-{\;C}_{e}\right)}{W}\;\text{×}\;V$$

(3) $$q_{t}=\frac{\left(C_{i}-{\;C}_{t}\right)}{W}\;\text{×}\;V$$

where q_e_ and q_t_ are the adsorption capacities of paraquat at equilibrium and time t (mg·g^−1^), respectively. C_0_, C_e_, and C_t_ are the initial, equilibrium, and time t paraquat concentrations in the solution (mg·L^−1^), respectively. V is the solution volume (L) and W is the weight of the material (g) [40].

#### 2.4.3. Adsorption Isotherm

##### Langmuir Isotherm

The Langmuir model assumes that the maximum adsorption capacity corresponds to a monolayer of adsorbate molecules on the adsorbent surface. It is also assumed that adsorbate molecules bind to specific sites and each site accommodates one molecule. It is further assumed that the adsorptive energy is equal for all sites, regardless of the adsorbed molecules in neighboring sites. The adsorbent surface is flat, smooth, and adsorbate-adsorbate interactions are negligible. Equation (4) describes the Langmuir model

(4) $$q_{e}=\frac{q_{m}k_{L}C_{e}}{{1+k}_{L}C_{e}}$$

where q_e_ represents the paracetamol adsorption quantity, m is the maximum adsorption capacity and corresponds to the monolayer, C_e_ is the paracetamol concentration in equilibrium and k_L_ is the Langmuir constant [10].

##### Freundlich Isotherm

The Freundlich model is an empirical model that is frequently used to describe organic compound adsorption in an aqueous solution. It proposes an exponential decrease in the distribution of the active sites’ adsorption energies. The equation uses mathematical model (5)

(5) $$q_{e}{=k}_{F}C_{e}^{\frac{1}{n}}$$

where the constants K_F_ and n depend on the adsorbent–solute interaction and the temperature. The 1/n values may be less or greater than the unity. When the value is less than unity, this indicates favorable adsorption. This expression reduces to a linear adsorption isotherm. Normal adsorption occurs when the value of 1/n is below one. Cooperative adsorption arises in the case 1/n being above one [41].

##### Temkin Isotherm

This isotherm contains a component that explicitly considers interactions between the adsorbent and the adsorbate. It assumes that the adsorption heat linearly varies with the degree of overlap. This equation, which was formulated in the case of the adsorption of gases on solids and transported to the liquid phase, is one of the only equations providing access to the variation in adsorption energy, which characterizes how the pollutants’ molecules are retained on the surface of the adsorbent. For an intermediate concentration range, this isotherm is reasonably used. As the adsorption heat is a function of the temperature of all molecules in the layer, the model predicts that it will fall linearly rather than logarithmically as coverage increases. The Temkin isotherm was calculated according to the following equations:

(6) $$q_{e}{=\mathrm{BlnK}}_{T}{+\mathrm{BlnC}}_{e}$$

(7) $$B=\frac{\mathrm{RT}}{b}$$

K_T_ is a Temkin isotherm equilibrium binding constant (L·g^−1^); b, Temkin isotherm constant; R, universal gas constant (8.314 J·mol^−1^·K^−1^); T, the temperature at 298 K; B is a constant related to the heat of sorption (J·mol^−1^) [42].

##### Jovanovic Isotherm

The Jovanovic model is based on the Langmuir model’s assumptions, as well as the potential of certain mechanical contacts between the adsorbent and the adsorbate. The Jovanovic isotherm was calculated according to the following equations:

(8) $$q_{e}{=q}_{m}\left(1\;-{\;\exp}^{-{\mathrm{kjC}}_{e}})\right.$$

where q_e_ the is amount of adsorbate in the adsorbent at equilibrium (mg·g^−1^), q_m_ is maximum uptake of adsorbate, and kj is Jovanovic constant [43].

#### 2.4.4. Adsorption Kinetics

##### Pseudo-First-Order

The expression of the pseudo-first-order reaction model for *n* = 1 is as follows:

(9) $$q_{t}{=q}_{e}\left(1\;-{\;\exp}^{-k_{1}t}\right)$$

where q_e_ and q_t_ are the amounts of adsorbate uptake per adsorbent mass at equilibrium and at any time t (min), respectively, and k_1_ (min^−1^) is the rate constant of the PFO equation.

##### Pseudo Second Order

The expression of the pseudo-second-order reaction model for *n* = 2 is as follows:

(10) $$q_{t}=\frac{q_{e}^{2}k_{2}t}{q_{e}k_{2}t+1}$$

where q_e_ (mg·g^−1^) and q_t_ (mg·g^−1^) are the adsorbate amounts adsorbed at equilibrium and any t (min), respectively and k_2_ (g·mg^−1^·min^−1^) is the PSO equation constant rate.

##### Elovich

Elovich kinetic model is usually used in a gas–solid system and is expressed by:

(11) $$q_{t}=\frac{1}{\beta }\ln\left(1+\alpha \beta t\right)$$

where q_t_ is the amount of paraquat adsorbed in mg·g^−1^ at a particular time, t. α represents the initial adsorption rate in mg·g^−1^·min, and β is the extent of surface coverage in g·mg^−1^ and the process activation energy [43].

##### Inter-Particle Diffusion

The intraparticle diffusion model, which considers pore diffusion, was developed and proposed as follows:

(12) $$q_{t}{=k}_{\mathrm{diff}}t^{\frac{1}{2}}+C$$

where C (mg·g^−1^) is the intercept and k_diff_ (mg·g^−1^·min) is the intraparticle diffusion rate constant.

The internal diffusion model assumes that the internal diffusion of the adsorbate is the slowest step, resulting in the rate-controlling step during the adsorption process, and that adsorption is instantaneous [44].

## 3. Results and Discussion

### 3.1. Morphology and Characteristics

#### 3.1.1. Surface Morphology

**Figure 2:**
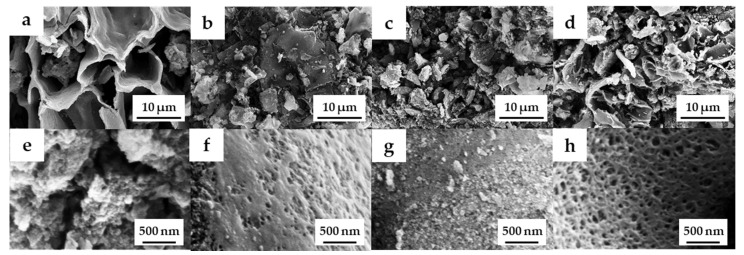
FE-SEM images of (**a**,**e**) OPL−HTC−800, (**b**,**f**) OPL−KOH−800−1:0.25, (**c**,**g**) OPL−KOH−800−1:1, and (**d**,**h**) OPL−KOH−800−1:4.

**Figure 3:**
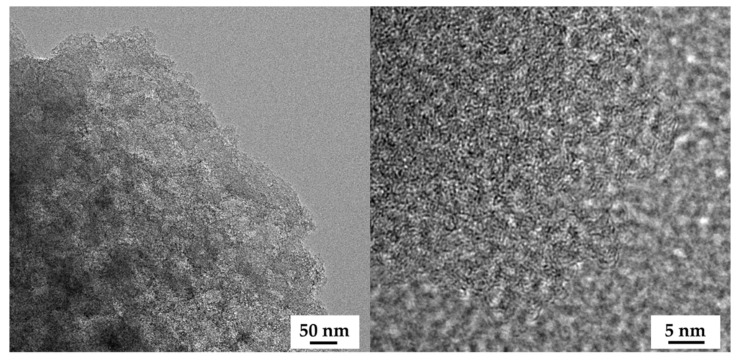
FE-TEM images of OPL−KOH−800−1:4.

The field emission scanning electron microscopy (FE-SEM) images of the OPL nano-porous carbon without and with KOH activation are demonstrated in Figure 2a–h. The surface of OPL−HTC−800 revealed a wavy and wrinkled morphology (Figure 2a,e), as opposed to the uniform, rough-surface morphology of the OPL−KOH−800 samples (Figure 2c,d), which contributed to its large surface area. This result confirms that KOH activation had a significant influence on the transformation of a non-porous structure to a well-developed porous sample. The activation at a high KOH content could enlarge the pore cavity size of a sponge-like morphology. As illustrated in high-magnification FE-SEM images (Figure 2f–h), the sponge-like structure of all the OPL nano-porous carbon samples was quite smooth, with significant differences when the KOH content increases. As demonstrated in Figure 3, transmission electron microscopy (TEM) revealed the presence of a nano-porous structure (micropores and mesopores). OPL−KOH−800−1:4 is an amorphous phase, which showed a nano-porous structure. The number of white dots in the carbon matrix between the disordered carbon layers suggests the presence of micropores and mesopores in OPL−KOH−1:4 (black).

#### 3.1.2. Nitrogen Adsorption/Desorption Isotherms and Pore Size Distribution

**Figure 4:**
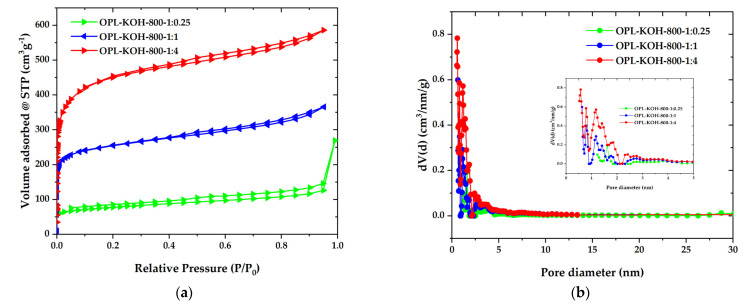
(**a**) N_2_ adsorption–desorption isotherms. Solid and open symbols represent the adsorption and desorption data, respectively. (**b**) DFT pore size distribution of OPL−KOH−800−1:0.25, OPL−KOH−800−1:1, and OPL−KOH−800−1:4.

To further examine the textural properties on a porous structure, the N_2_ adsorption-desorption isotherms were recorded and are illustrated in Figure 4a. The OPL−KOH−800−1:0.25 exhibited low quantities of N_2_ being adsorbed with a type-IV isotherm and H3 hysteresis loop, according to the IUPAC classification, which is characteristic of mesoporous materials. The mesoporous structure of OPL−KOH−800−1:0.25 could be attributed to the slit-shaped pores that were likely created by the spaces between the wavy edges on the surface. For the OPL-KOH samples, it is evident that the quantities of adsorbed N_2_ increased as the KOH content increased, implying that the surface area was enhanced. The specific surface area was determined by the Brunauer–Emmett–Teller (S_BET_) method, using adsorption data in the relative pressure range of 0.05–0.30. After the KOH activation, S_BET_ increased to 283, 961, and 1681 m^2^·g^−1^ for OPL−KOH−800−1:0.25, OPL−KOH−800−1:1, and OPL−KOH−800−1:4, respectively. Meanwhile, the isotherms OPL−KOH−800−1:1 and OPL−KOH−800−1:4 exhibited a mixed type-I/type-IV isotherm with a narrow pore, showing that a highly microporous character existed. However, as the KOH content increased, a wider isotherm pore and a narrow hysteresis loop with an H4 type (P/P0 = 0.40–0.99) were observed, resulting in the formation of larger micropores and the development of mesopores. Figure 4b illustrates the pore size distribution of the samples as determined using the density functional theory (DFT) method. The majority of OPL−KOH−800−1:0.25, OPL−KOH−800−1:1, and OPL−KOH−800−1:4 pores were found in the micropore region (<2 nm). Following activation at higher KOH contents, the micropores were widened into small mesopores (2–5 nm). From the t-plot analysis, the proportion of the external surface area caused by the meso-macropores increased from 126 m^2^·g^−1^ for OPL−KOH−800−1:0.25, 269 m^2^·g^−1^ for OPL−KOH−800−1:1, and 404 m^2^·g^−1^ for OPL−KOH−800−1:4. The total pore volume (V_total_) increased from 0.845 to 0.907 cm^3^·g^−1^. The textural parameters of all samples are summarized in Table 2. These results indicate that chemical activation with a high KOH content delivered a significant increase in S_BET_ and further meso-porosity development, which agree with the previously reported observation describing the effect of KOH activation on the surface area and pore structure of OPL−KOH [45,46,47]. Table 3 displays the activated carbon produced by different manufacturing processes and different conditions compared to this study.

#### 3.1.3. Crystallinity

**Figure 5:**
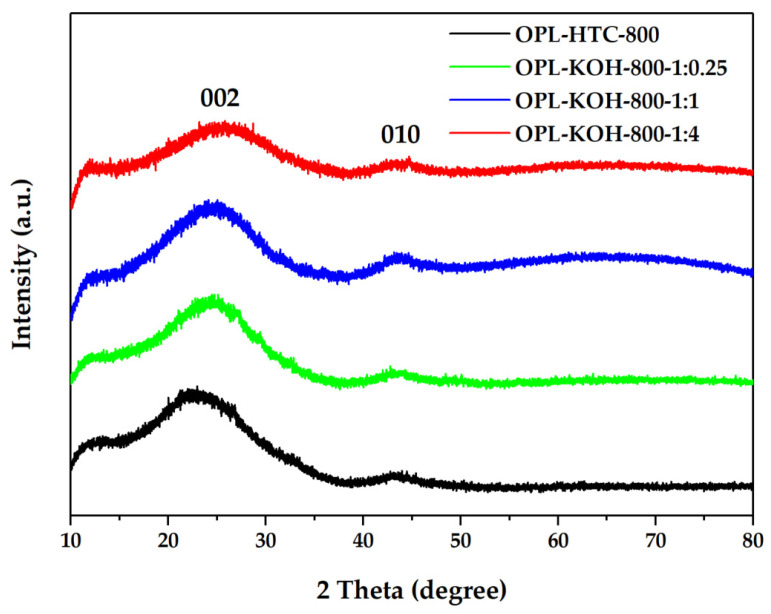
XRD patterns of OPL−HTC−800, OPL−KOH−800−1:0.25, OPL−KOH−800−1:1, and OPL−KOH−800−1:4.

From Figure 5, The OPL−HTC−800, OPL−KOH−800−1:0.25, OPL−KOH−800−1:1, and OPL−KOH−800−1:4 sample. The XRD measurement at a range of 10° < 2*θ* < 80° was investigated to examine the phase structure of the carbon material, as shown in Figure 5. The peak at a 2*θ* around 19−26° showed carbon characteristics in an amorphous phase. In addition, the peak found at 2*θ* positions of 25.5° and 43° correspond to the structure of carbon materials, as shown in Figure 5. The 002 peaks in crystalline graphite occurred at 23° and the broad 101 peaks appeared to be a single peak. This implied that each carbon atom layer in the structure was incompletely stacked [46,47]. These results prove that all the samples consist of small sp^2^ platelets. The first peak corresponded to the reflection of graphite and the stacking of graphene layers. The other peaks were also ascribed to the reflections originating from the in-plane structure of graphitic in the structure of corresponding samples.

#### 3.1.4. Function Groups

**Figure 6:**
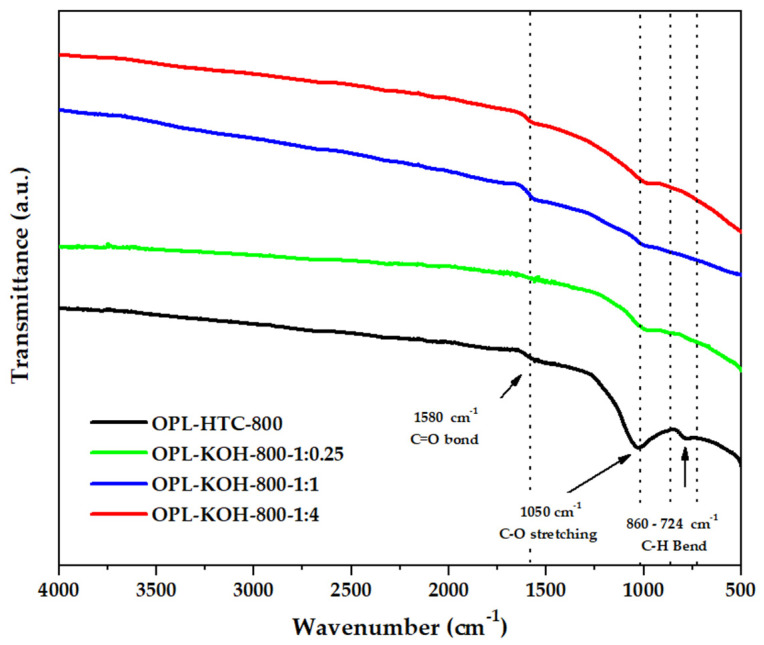
FTIR spectra of OPL−800, OPL−KOH−800−1:0.25, OPL−KOH−800−1:1, and OPL−KOH−800−1:4.

The chemical functionality of all samples could be revealed by analyzing their FTIR spectra in Figure 6. The FTIR spectra of porous carbon synthesized from palm leaves showed that the peaks at and 1580 cm^−1^ represent the C=O bonds of the carboxylic groups (−COOH) and the stretching vibrations of conjugated C–C bonds of aromatic rings, whereas those of C=C and C–O stretching (1050 cm^−1^) remained following the emergence of C=C bending (860–724 cm^−1^), described as hemicellulose. This result suggests that KOH activation at high temperatures promoted the degradation of the lignocellulose structure and the removal of functional groups to yield products with a high carbon content [48].

**Figure 7:**
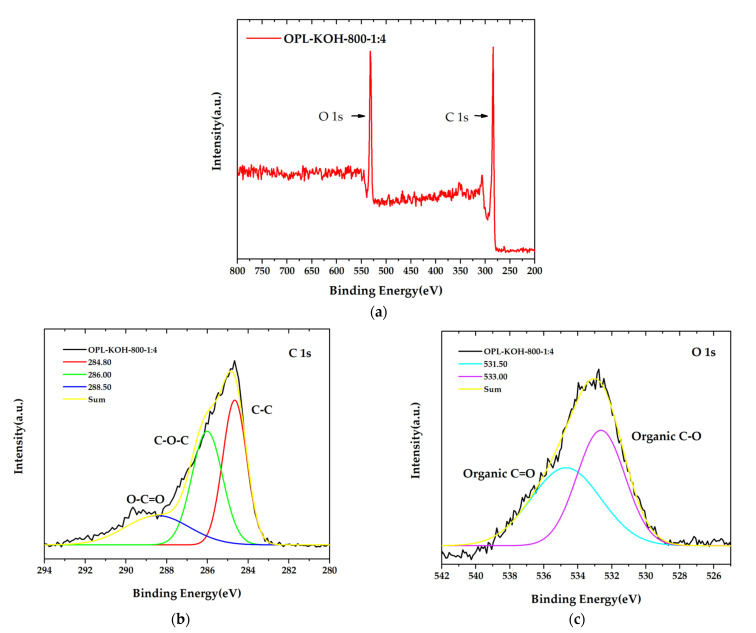
X-ray photoelectron spectroscopy (XPS) spectra of OPL−KOH−800−1:4 (**a**) the main XPS spectra, (**b**) C1s spectra peak, and (**c**) O1s spectra.

The surface chemistry composition of OPL−KOH−800−1:4 with the highest specific surface areas 1684.860 m^2^/g was accomplished using XPS analysis, as demonstrated in Figure 7. The obtained XPS spectra of prior to and after KOH activation samples can be fitted to two enriched component peaks in carbon and oxygen, as seen in Figure 7a. The high-resolution C 1s (Figure 7b) peak in OPL−KOH−800−1:4 can be separated into three component peaks, representing the peaks in graphitic carbon (C=C, 284.80 eV), the carbon groups in alcohol and/or ether linkages (C–O–C, 286.00 eV), and carbon in carbonyl group (O–C=O, 288.50 eV). The O 1s spectrum exhibits the two relevant spectra representing organic C–O in phenol and ether groups, and the organic C=O in carboxylic acid and/or ester groups, centered at 531.00 and 533.00 eV, respectively, as displayed in Figure 7c. Meanwhile, the O 1s of OPL−KOH−800−1:4 was fitted into two components: the organic C–O in phenol and ether groups of 531.00 eV, and organic C=O in carboxylic acid and/or ester groups of 533.00 eV. The results of XPS analysis could possibly be used to indicate the significantly different proportions of carbon and oxygen, which is good agreement with FTIR and ultimate analyses.

#### 3.1.5. Amorphous Structure

**Figure 8:**
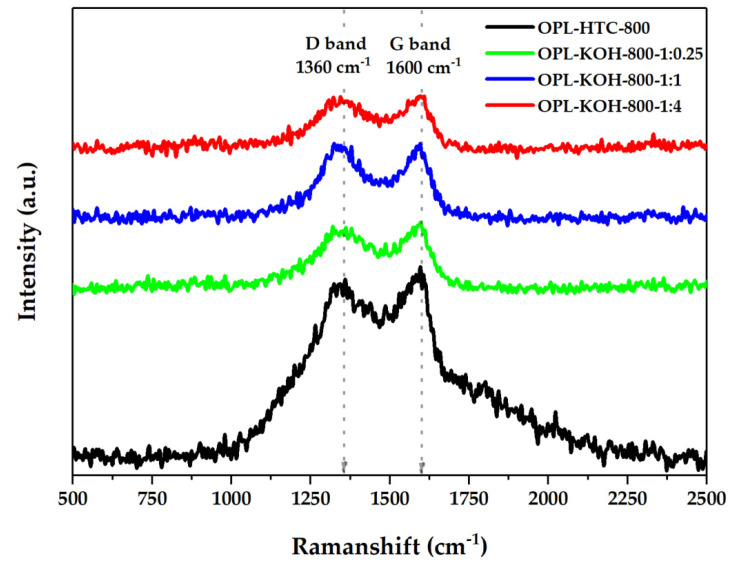
Raman spectra of OPL−HTC−800, OPL−KOH−800−1:0.25, OPL−KOH−800−1:1, and OPL−KOH−800−1:4.

The characterization of the chemical properties of OPL−KOH is illustrated in Figure 8. The crystallography of the OPL−KOH sample (Figure 8) was evaluated using the Raman technique from I_D_/I_G_ ratio, considering amorphous and crystallite carbon components. The band at 1360 cm^−1^ was assigned to the D band, which corresponds to a graphitic lattice vibration mode with A1g symmetry. This is typical for carbon materials, which was detected to be around 1300–1380 cm^−1^. The G band, which typically occurs at 1600 cm^−1^, arises from the stretching of the C–C bond in graphitic materials, and is common for all sp^2^ carbon systems. Hence, I_D_/I_G_ shows (Table 4) the amorphous ratio that represents the physical structure of porous carbon. The I_D_/I_G_ values of the OPL−HTC−800, OPL−KOH−800−1:0.25, OPL−KOH−800−1:1 and OPL−KOH−800−1:4 was 0.96, 0.96, 0.97 and 0.99, respectively. The Raman analyzes reveal that OPL−KOH−800−1:4 contributes slightly more to the graphitic structures [35].

#### 3.1.6. The Ultimate Analysis

The elemental compositions of OPL-KOH-800-1:4 is summarized in Table 5. The C, H, O, and N contents of OPL−KOH−800−1:4 is listed in Table 5. The C content markedly increased, from 46.204% to 77.860%, the H content increased from 2.355% to 5.699%, and the N content increased from 0.084% to 1.748%. However, the O content decreased from 46.349 % to 16.997%. This result demonstrates that carbon and oxygen functional groups were introduced to the surface of OPL-KOH-800-1:4.

### 3.2. Adsorption Property of Materials

#### 3.2.1. The Removal Efficiency

**Figure 9:**
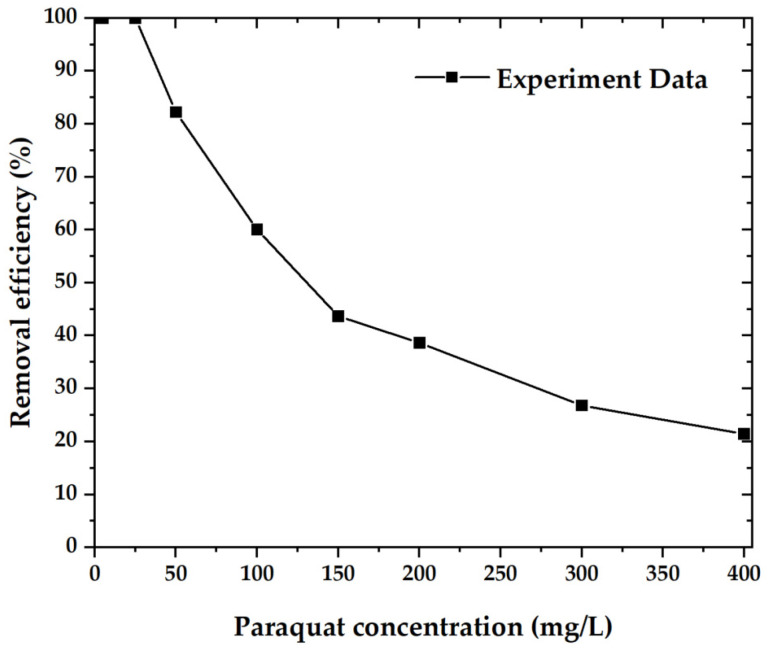
The removal efficiency of the material with paraquat.

Figure 9 shows the paraquat removal efficiency of OPL−KOH−800−1:4 samples, which are plotted as a function of paraquat concentration between 5 and 400 mg·L^−1^. In descending order, the paraquat concentration reached 100% removal efficiency up to a concentration of 25 mg·L^−1^ and dropped to 21.42% at a concentration of 400 mg·L^−1^ [35,36,37].

#### 3.2.2. Adsorption Isotherms

**Figure 10:**
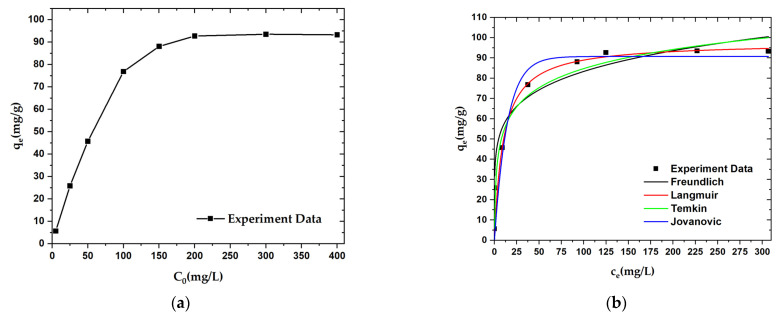
Adsorption curves for paraquat adsorption onto OPL−KOH−800−1:4: (**a**) effect of initial concentration on the adsorption capacity; (**b**) adsorption isotherm curves.

An adsorption isotherm experiment was carried out to explore the adsorption mechanism for OPL−KOH−800−1:4 on paraquat. The adsorption capacity of OPL−KOH−800−1:4 was found to increase from 5.681 mg·g^−1^ to 93.306 mg·g^−1^ with increases in initial paraquat concentration from 5.0 mg·L^−1^ to 400.0 mg·L^−1^. When the paraquat concentration was further increased to 400.0 mg·L^−1^, the adsorption capacity remained almost unchanged at a value of 93.306 mg·g^−1^ (Figure 10a). According to the apparent area of the paraquat, the external and internal surfaces of the OPL−KOH−800−1:4, and the maximum adsorption capacity, we can estimate that most of the paraquat is adsorbed onto the pores of the nano-porous carbon. The equilibrium isotherm data were fitted with Langmuir, Freundlich, Temkin, and Jovanovic isotherm models, respectively, and the fitting curves for all four models are shown in Figure 10b. Compared with the four fitting results, only the correlation coefficient value (R^2^) was found to change. The R^2^ values suggest that the data for paraquat adsorption onto OPL-KOH-800-1:4 are best matched with the Langmuir isotherm (Table 6). The Langmuir isotherm is a typical monolayer adsorption model and is suitable for describing an adsorption process without intermolecular interaction. The calculated maximum adsorption capacity (q_m_) for paraquat (97.755 mg·g^−1^) using the Langmuir model was found to be higher than that of most other adsorbents. Nonetheless, high alkalinity tends to increase the adsorptive properties of paraquat due to the surface characteristics of porous carbon.

#### 3.2.3. Adsorption Kinetics

**Figure 11:**
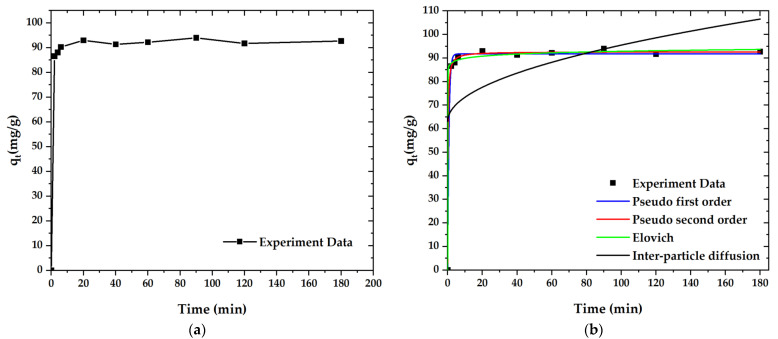
Adsorption curves for paraquat adsorption onto OPL−KOH−800−1:4: (**a**) effect of adsorption time on the adsorption capacity; (**b**) curves of the adsorption kinetics.

The kinetics’ fitting curves and corresponding parameters are shown in Figure 11. According to the non-linearity (R^2^), the adsorption processes of paraquat (R^2^ = 0.999) is closely approximated to the pseudo-second-order kinetics model. These results further confirm that the adsorption mechanism of paraquat on OPL-KOH-800-1:4 is mostly chemical adsorption (Table 7). Technically, the adsorption in higher-temperature condition exhibited an increase in adsorption rate. This is due to the shaking of adsorbate molecules, which caused the turbulent flow, resulting in an enhancement of adsorption performance.

#### 3.2.4. Comparative Profile

To evaluate the development behavior of the new adsorbent proposed in this research, a comparison study was performed between the nanoporous of the oil palm leaves and other adsorbents reported in previous studies (Table 8). Notably, oil palm leaves biomass has excellent adsorption capacity for paraquat and a fast adsorption process, as it reaches equilibrium in a few minute, standing out in relation to other bioadsorbents found in the recent literature.

### 3.3. Regeneration Efficiency

**Figure 12:**
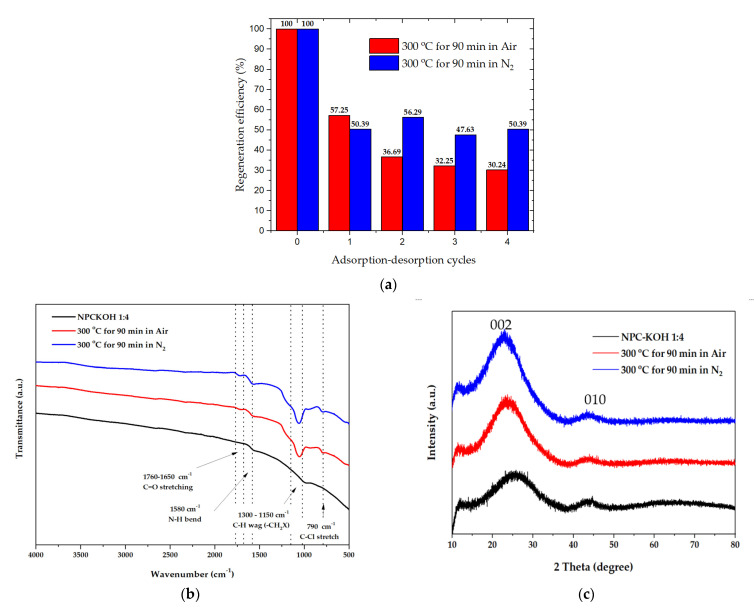
(**a**) Regeneration efficiency of the material with paraquat after four adsorption–desorption cycles.; (**b**) FTIR spectra of the material with paraquat after four adsorption–desorption cycles.; (**c**) XRD patterns of the material with paraquat after four adsorption–desorption cycles. The concentration of paraquat solution was 200 mg·L^−1^.

A material’s regeneration ability is important for practical applications [39,55]. The target-loaded materials were treated with pyrolysis at 300 °C at 90 min with nitrogen flow and pyrolysis at 300 °C at 90 min in airflow to remove the targets [46]. The materials were then washed, dried, and subjected to the next round of adsorption experiments. The materials retained more than 50.39 to 47.63% of pyrolysis at 300 °C for 90 min in nitrogen flow, and pyrolysis at 300 °C at 90 min in nitrogen flow. The retained materials gradually decreased from 100 to 47.63% of their initial adsorption capacity after four adsorption–desorption cycles (Figure 12a).

The chemical functionality with paraquat after four adsorption–desorption cycles could be revealed by analyzing the FTIR spectra in Figure 12b. The FTIR spectra of porous carbon synthesized from palm leaves showed that the peaks at 1700–1580 cm^−1^ represented the C=O bonds in the carboxylic groups (−COOH) and the stretching vibrations of conjugated C–C bonds of aromatic rings, whereas for those at the N-H bend (1580 cm^−1^) of the 1^o^ amines, the peaks at 1300–1150 cm^−1^ represented C-H wag (−CH_2_X) and the peaks at 790 cm^−1^ represented H-Cl’s correlated with alkyl halides. This suggests that the paraquat is completely adsorbed on the carbon surface, after several adsorption–desorption tests. These functional groups persisted, resulting in a decrease in adsorption capacity.

The XRD measurement at the range of 10° < 2*θ* < 80°, was investigated to examine the phase structure of carbon material, as shown in Figure 12c. The peak of 2*θ* at around 19−26° showed the carbon characteristics in an amorphous phase. In addition, the peak found at 2*θ* in positions of 25.5° and 43° was assertive for all samples corresponding to the carbon materials. As shown in Figure 12c, the 002 peaks in crystalline graphite occurred at 23° and the broad 101 peaks appeared to be a single peak. This can be implied that the structure of OPL nano-porous carbon has transformed into amorphous phase after regeneration because of the effect of pyrolysis condition.

## 4. Conclusions

Nano-porous carbon was prepared from oil palm leaves via hydrothermal carbonization at 200 °C for 12 h under a nitrogen atmosphere. It was stimulated with 1:4 potassium hydroxide at 800 °C for 1 h under a nitrogen atmosphere. According to KOH activation, OPL samples showed a porous, sponge-like morphology with a large surface area. This result is due to an increase in the specific surface area and volume of the pore, caused by the microporous pore structure developing into a hierarchical mesoporous. The increased removal efficiency and maximum adsorption capacity of carbon nano-cavities can be attributed to the large surface area and microporous and mesoporous pore sizes. In addition, the carbon nanotubes obtained from this research can be used as an alternative adsorbent to remove paraquat and other organic compounds from water. The adsorption isotherm models of OPL−KOH−800−1:4 was represented by the Langmuir, Freundlich, Temkin, and Jovanovic isotherm models. Their adsorption kinetics could be described by the Langmuir model. The results of the adsorption isotherm and kinetics models confirm that the adsorption mechanisms of paraquat were a typical monolayer adsorption model and chemical adsorption. The regeneration ability of the material further shows great application potential for controlling water pollution.

## Figures and Tables

**Table 1:** The ultimate analysis of oil palm leave (OPL).

Condition	Ultimate Analysis **			
	C	H	N	O *
OPL	42.264	6.309	1.377	50.050

* Calculated by different, ** (as-received basis, *w*/*w*).

**Table 2:** Structural parameters of OPL−KOH−800−1:0.25, OPL−KOH−800−1:1, and OPL−KOH−800−1:4 from N_2_ adsorption.

Condition	^1^ S_BET_ (m^2^·g^−1^)	^2^ Micropore Surface Area (m^2^·g^-1^)	^3^ External Surface Area (m^2^·g^−1^)	^4^ Vtotal (cm^3^·g^–1^)	^5^ D_mic_ (nm)	^6^ D_mes_ (nm)	^7^ D_average_ (nm)
OPL−KOH−800−1:0.25	283	156	126	0.845	1.231–1.931	2.020–13.376	11.96
OPL−KOH−800−1:1	961	692	269	0.566	0.548–1.931	2.020–13.376	2.36
OPL−KOH−800−1:4	1685	1281	404	0.907	0.523–1.931	2.020–13.376	2.15

^1^ S_BET_: the specific surface area is calculated by Brunauer–Emmett–Teller (BET) model; ^2^ Micropore surface area and ^3^ External surface area: the specific surface area is calculated by V-t method; ^4^ V_total_: the total pore volume is calculated by Barrett–Joyner–Halenda (BJH) model; ^5^ D_mic_ and ^6^ D_mes_: the micropore and mesopore size distribution are obtained by Density Functional Theory (DFT) model; ^7^ D_average_: the average pore size distribution is calculated by Density Functional Theory (DFT) model.

**Table 3:** Production of activated carbon by different manufacturing processes and under different conditions.

Sample	Condition	S_BET_ (m^2^·g^−1^)	Vtotal (cm^3^·g^−1^)	D_average_ (nm)	Ref
Onion Leaves (*Allium fistulosum*)	Chemical activation process using (H_3_PO_4_)	1100	0.879	1.050	[41]
Biochar derived from tobacco stems	Stems and pyrolyzed 400 °C for 30 min	33	0.072	1.631	[37]
Bamboo shoot shell	(HTC) 800 °C	513	0.27	2.09	[5]
Oil Palm Male Flowers	Microwave-Assisted Pyrolysis Combined KOH Activation	991	0.49	-	[35]
Water ferns	(HTC) followed by a chemical activation process using (KOH) at 700 °C	2848	1.552	-	[20]
Oil palm leave	(HTC) followed by a chemical activation process using (KOH) at 700 °C	1685	0.907	2.15	This study

**Table 4:** Raman spectra of OPL via hydrothermal carbonization and KOH activation.

Condition	I_D_	I_G_	I_D_/I_G_
OPL−HTC−800	157.10	162.42	0.96
OPL−KOH−800−1:0.25	55.12	57.28	0.96
OPL−KOH−800−1:1	64.46	65.78	0.97
OPL−KOH−800−1:4	49.11	49.49	0.99

**Table 5:** Ultimate analysis of OPL via hydrothermal carbonization and KOH activation.

Condition	Ultimate Analysis **			
	C	H	N	O *
OPL−HT−200−12	46.204	5.699	1.748	46.349
OPL−HTC−800	72.296	2.355	0.841	24.508
OPL−KOH−800−1:0.25	73.352	2.742	1.350	22.556
OPL−KOH−800−1:1	75.338	4.188	0.590	19.884
OPL−KOH−800−1:4	77.860	5.059	0.084	16.997

* Calculated by different, ** (as-received basis, *w*/*w*).

**Table 6:** Adsorption isotherm parameters of paraquat.

Model	Parameter	Paraquat
Langmuir $q_{e}=\frac{q_{m}k_{L}C_{e}}{{1+k}_{L}C_{e}}$	q_m_ (mg·g^−1^)	97.755 ± 7.058
	k_L_ (L·mg^−1^)	0.100 ± 0.047
	R^2^	0.916
Freundlich $q_{e}{=k}_{F}C_{e}^{\frac{1}{n}}$	$k_{F}$	38.615 ± 7.532
	n	5.986 ± 0.039
	R^2^	0.906
Temkin $q_{e}{=\mathrm{BlnK}}_{T}{+\mathrm{BlnC}}_{e}$ $\;;\;B=\frac{\mathrm{RT}}{b}$	B	13.621 ± 4.082
	$K_{T}$	5.034 ± 9.253
	R^2^	0.898
Jovanovic $q_{e}{=q}_{m}\left(1-{\;\exp}^{-{\mathrm{kjC}}_{e}})\right.$	$q_{m}$	90.764 ± 5.572
	k	0.069 ± 0.025
	R^2^	0.906

Note: T(K) is the temperature in Kelvin; R is the ideal gas constant (8.314 J·mol^−1^·K^−1^); K_L_, K_F_, n, B, K_T_, k and b are the constants for the corresponding formula, respectively.

**Table 7:** Adsorption kinetic parameters of paraquat.

Model	Parameter	Paraquat
Pseudo-first-order $q_{t}{=q}_{e}\left(1-e^{-k_{1}t}\right)$	k_1_ (min^−1^)	1.397 ± 0.153
	q_e_ (mg·g^−1^)	91.774 ± 0.581
	R^2^	0.997
Pseudo-second-order $q_{t}=\frac{q_{e}^{2}k_{2}t}{q_{e}k_{2}t+1}$	k_2_ (min·mg·g^−1^)	0.069 ± 0.010
	q_e_ (mg·g^−1^)	92.673 ± 0.374
	R^2^	0.999
Elovich $q_{t}=\frac{1}{\beta }\ln\left(1+\alpha \beta t\right)$	α	4.343 × 10^29^ ± 6.465 × 10^30^
	β	0.781 ± 0.166
	R^2^	0.998
Inter-particle diffusion $q_{t}{=k}_{\mathrm{diff}}t^{\frac{1}{2}}+c$	k_diff_ (mg·g^−1^·min^−1/2^)	3.236 ± 1.959
	c (mg·g^−1^)	63.143 ± 14.155
	R^2^	0.254

Note: k_1_, k_2_, α, β, $k_{\mathrm{diff}}$ and c are the constants for the corresponding formula, respectively.

**Table 8:** Comparison of q_m_, k_L_, concentration and dosage values from the Langmuir isotherm model of adsorbents and modified adsorbents for the removal of paraquat.

Sample	q_m_ (mg·g^−1^)	k_L_ (L·mg^−1^)	Paraquat Concentration (mg·L^−1^)	Dosage (g·L^−1^)	Ref
Water ferns	5.78	0.26	1.5–4.5	1.00	[20]
NAC water ferns	20.00	1.39	1.5–45	1.00	[20]
Mesoporous silica	11.75	1.19	8–16	0.04	[28]
Poly(Vinyl Alcohol)- Cyclodextrin	102.00	0.09	25–300	2.00	[49]
Starch-derived carbons	66.20	25.61	1–150	2.00	[50]
Carbon tubes	218.61	0.03	70–500	0.20	[19]
Magnetic adsorbent	242.40	0.66	30–900	2.50	[26]
TEMPO-oxidized cellulose nanofibers	115.00	3.51	10	0.10	[51]
Bentonite	94.34	480.83	50	2.00	[52]
K-Zeolite LTL	166.71	1.05	50–500	2.50	[53]
H-Zeolite LTL	25.67	1.26	50–500	2.50	[53]
NaY zeolite	234.40	0.05	100–1500	2.50	[54]
Nanoporous for Oil palm leave	97.76	0.10	25–400	1.00	This study

## Data Availability

Not applicable.
